# Case report and literature review: Treatment of multiple meningiomas combined with multiple unruptured aneurysms in a single operation

**DOI:** 10.3389/fsurg.2022.971068

**Published:** 2022-09-07

**Authors:** Ren-jie Wei, Xiao-lin Wu, Feng Xia, Jing-cao Chen

**Affiliations:** ^1^ Department of Neuro-Surgery, Zhongnan Hospital of Wuhan University, Wuhan, China; ^2^ Department of Hepatic Surgery, Tongji Hospital of Tongji Medical College of Huazhong University of Science and Technology, Wuhan, China

**Keywords:** falx brain tumor, multiple aneurysms, case report, imaging, multiple meningiomas

## Abstract

**Background:**

Although the incidence of a single meningioma or a single aneurysm is common, cases of multiple meningiomas combined with multiple aneurysms are rarely reported, and surgical treatment of the coexisting situation is also relatively uncommon.

**Case presentation:**

A 38-year-old male patient presented to the neurosurgery department of our center with a headache. Examination revealed only symptoms of headache. Laboratory tests showed only decreased total protein and albumin. Magnetic resonance imaging showed preoccupation with the frontal lobe and the right temple bone. Magnetic resonance angiography and digital subtraction angiography showed two aneurysms in the anterior communicating artery and right anterior cerebral artery. Based on a combination of the patient’s history and imaging, we hypothesized that the patient was simultaneously suffering from meningioma and an aneurysm, and both of them are multiple. The patient underwent tumor resection and clipping procedure based on this hypothesis in one surgery. Intraoperative biopsy proved to be a meningioma. The patient was discharged on the 10th postoperative day, and a postoperative follow-up suggested no complications.

**Conclusion:**

Multiple meningiomas combined with multiple aneurysms are rare to be reported in the same patient. For those unruptured intracranial aneurysms (UIAs) located in the visual field of craniotomy prepared for brain tumorlike meningioma, it is possible to do the clipping as well. When the meningiomas are multiple, fitted with the surgical indication, and located in a position that cannot be treated in one surgery, this may lead to a two-stage operation, no matter where the UIAs are located.

## Introduction

Although intracranial tumor seems to have totally different pathogenesis from cerebral vascular diseases like aneurysm, there are still scattered reports about the coexistence of meningiomas and aneurysms (1–10). The incidence of meningioma is about 0.5% (8). The rate of occurrence of multiple meningiomas is 8%. The existence of multiple meningiomas and multiple aneurysms is even rarer to be reported.

Here, we treat a patient with three meningiomas and three aneurysms, and we discuss the etiology based on the clinical and histological findings.

## Method

A literature review was performed. The PubMed database was screened for meningioma and aneurysm cases from 1997 to 2022 according to the PRISMA using the following key terms: “meningioma and aneurysm,” “meningioma co-exist with aneurysm,” and “multiple meningiomas and multiple aneurysms (Table 1).”

**Table 1:** Summary of nine cases with coexisting meningiomas and aneurysms.

Case series	Age/sex	Clinical symptoms	Location of aneurysms	Location of meningiomas	Surgery	Follow-up
Javalkar (2009)	70/F	Headache diminished vision in the right eye	Left and right PCOM	Right pterional	Excision/clipping	Good
Tachikawa (2002)	51/M	Generalized seizure	Anterior ethmoidal artery	Olfactory groove	Excision	—
Takeda (2017)	75/F	Visual impairment of the left eye	ICPC aneurysm	Parasellar	Excision/clipping	Good
Kanamori (2013)	64/F	Headache and nausea	ICPC aneurysm	Left petrous	Clipping	—
Stevenson (1994)	48/M	Rotational vertigo impaired consciousness and frontal headache	Right middle cerebral artery, right pericallosal artery, and left ophthalmic artery	Right pterional and right parasagittal	Excision/clipping	Good
Tanaka (2022)	52/F	Headache	left ophthalmic artery	Right frontal lobe	Excision/embolization	Good
Ogino (1999)	70/F	Headache and nausea	ACOA (inside of tumor)	Sellae	Excision/clipping	Bilateral anosmia hydrocephalus
Dolenc (1998)	50/M	Right eye vision loss	ACOA	Sellae	Excision	Good
Current study	38/M	Headache	ACOA/right A2 right A3	Falx frontal skull base and right temple bone	Excision/clipping	Good

ACOA, anterior communicating artery; PCOA, posterier communicating artery; ICPC, internal carotid artery-posterier communicating artery.

### Case presentation

A 38-year-old man was admitted to the neurosurgery department of our center for treatment of headaches, complaining of a 1-week history of headaches. Prior to admission, a magnetic resonance imaging (MRI) scan had been performed at the previously visited hospital, and no invasive methods or medications were administered to the patient. On admission, we performed routine laboratory tests such as blood count, liver and kidney function, and coagulation. The patient had normal muscle strength, normal sensation, and good function of intracranial nerves on physical examination. Total protein was 63.9 g/L, and albumin was 38.5 g/L.

On the first day after admission, an MRI scan was performed on the patient.

**Figure 1:**
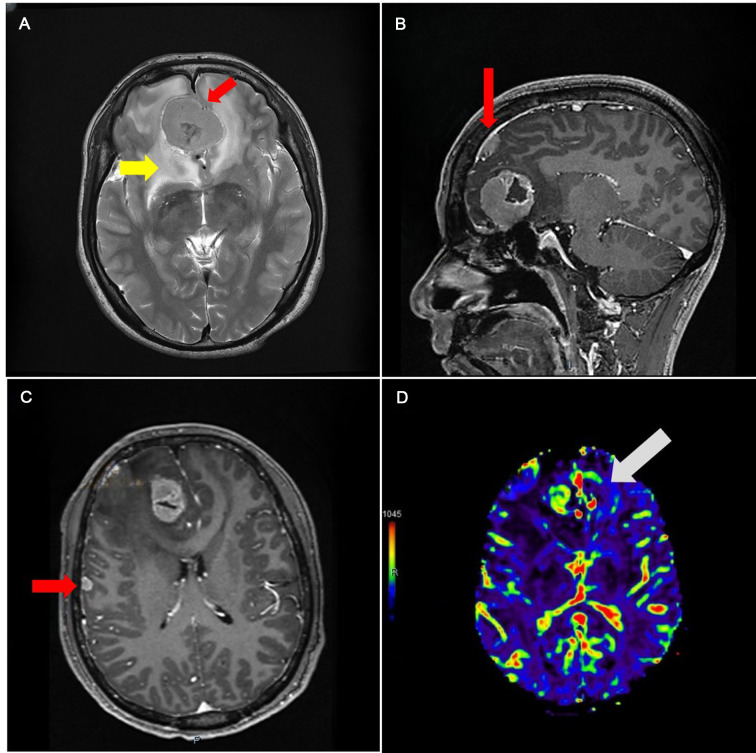
(**A**) Magnetic resonance image (MRI) image showing isointensity occupancy in T2 series with a clear border compared to the brain tissue (red arrowhead), surrounded by edema (yellow arrowhead). (**B**) Enhanced series showing the falx (red arrowhead). (**C**) Lesion with fair enhancement under the right temple bone. (**D**) CBV showing that the skull base area has more but not fair blood volume.

**Figure 2:**
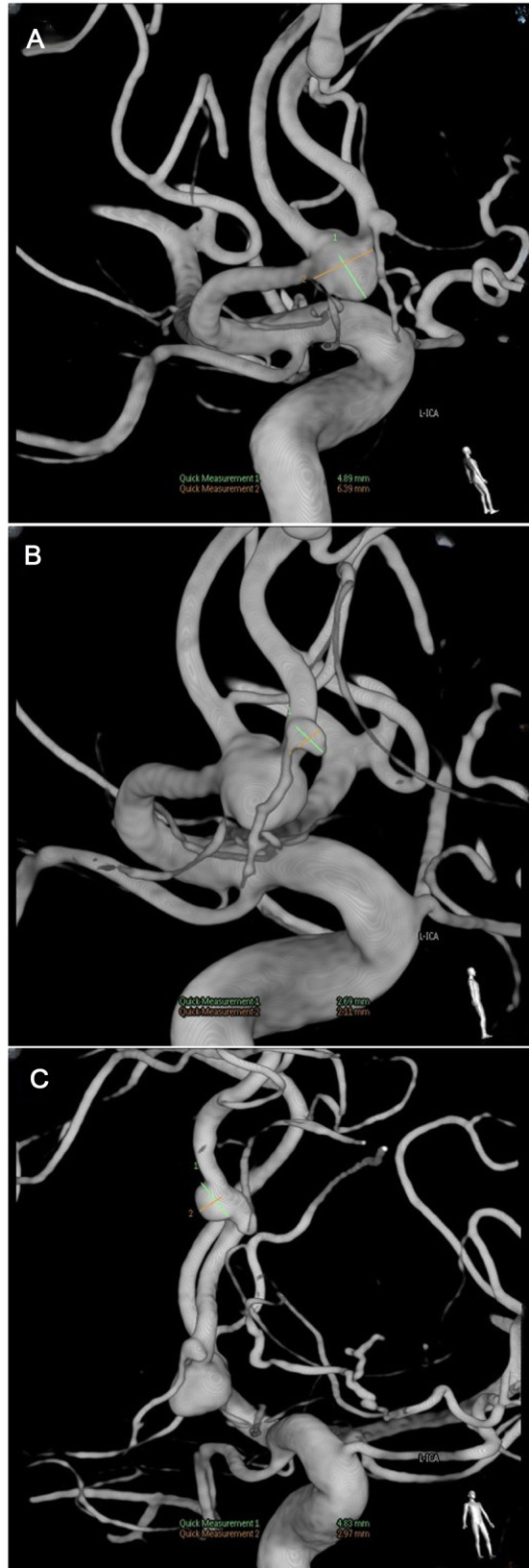
Digital subtraction angiography showing three aneurysms’ size and location.

**Figure 3:**
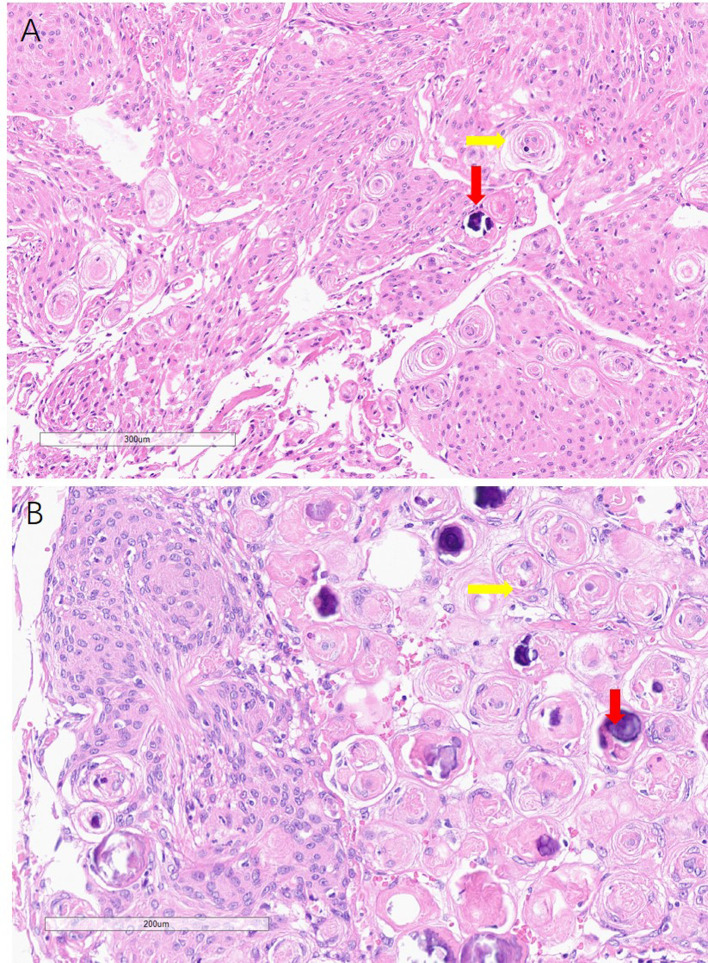
Histology of the meningiomas, showing psammoma bodies (yellow arrowhead) and calcification (read arrowhead) in both falx and skull base meningiomas.

The size of skull base cerebral occupancy was 41 × 31 mm, and the falx occupancy was 10 × 10 mm. The signal of two occupancies showed isointensity in both T1 and T2. The enhanced MRI report showed an unbalanced enhancement of the occupancy; the center of the occupancy was not enhanced (Figures 1–3). We also found a 7 × 6 mm enhancing node under the right temple bone.

Magnetic resonance spectroscopy shows that, according to the normal brain tissue, the Cr peak was slightly increased but the N-acetyl aspartate (NAA) peak was not remarkable. Perfusion weighted imaging shows that the occupancy has the feature of increased cerebral blood flow and cerebral blood volume (CBV) and delated mean transit time and time to peak (Figure 1). Magnetic resonance angiography (MRA) shows a cystic vascular change in both anterior communicating artery (ACOA) and right anterior cerebral artery (R-ACA) (A2), while the A1 portion of R-ACA does not appear to have this change.

The patient underwent a digital subtraction angiography (DSA) on the third day after admission, as the local hospital report mentioned an intracranial aneurysm. The result shows that three aneurysms are located in the ACOA and R-ACA (A2 + A3) (Figure 2).

Based on the patient's history, signs, symptoms, and supplement findings, we made a primary diagnosis of three occupancies, inferring them as meningiomas, but a definitive diagnosis would require biopsy results. The aneurysm was sure. According to the patient’s condition, a surgery plan was made. We decided to perform resection, clipping, and indocyanine green choroidal (ICG) angiography together.

On the sixth day after admission, we decided to perform tumor resection and aneurysm.

We exposed the brain tissue through an interhemispheric fissure approach, and a tumor of about 1 cm was seen besides the falx, with normal blood supply; we covered it with cotton pads, and then the frontal lobe near the midline was lifted to reveal the tumor at the anterior skull base; the tumor was attached to the anterior skull base and falx cerebral. The blood supply of the tumor surface was normal, the tumor texture was slightly benevolent, and the color was grayish white. Tumor surface had a capsule, the boundary with the surrounding normal brain tissue was clear, and the surrounding brain tissue was obviously edema. We freed the tumor along the tumor capsule and resected the skull base tumor.

After the resection of the skull base, we lifted the frontal lobe to expose the right frontal base, the A1 segment of the anterior cerebral artery, and the bilateral A2 segment; the anterior communicating artery was exposed along the direction of the interhemispheric fissure. The sizes of the aneurysms were 4 mm * 6 mm, 2 mm * 2 mm, and 4 mm * 2 mm. After the temporary aneurysm clip was used to block A1, the back of the neck of the aneurysm was fully dissociated, and three aneurysm clips were successfully applied to the anterior communicating aneurysm. The A2 and A3 segments of the aneurysm were successfully clipped with an aneurysm clip. Then, ICG fluoroscopy showed that the aneurysm was not developed, and the A1 and bilateral A2 arteries were developed smoothly.

Then, the right para-falx meningioma was resected and completely removed. The surgery was successful.

On the third postoperative day, an MRI review showed that the skull base and the falx occupancies had been resected, and computed tomography (CT) angiography showed that all three aneurysms had been successfully clipped.

The histologies of the skull base and falx occupancy were both reported to be endothelial meningiomas (Figure 3). The immunohistochemical technique shows EMA(+), GFAP(−), Ki-67(2%), PR(+), SSTR2(+), VIMENTIN(+).

The patient had minor epilepsy once on the third day after the surgery; sodium valproate was given, and epilepsy never happened again.

CT reviews of the brain and lungs were also performed three times on the first, third, and sixth postoperative days to check the bleeding and lesions. After the recovery time, the patient stated that the headache had disappeared.

Follow-up was planned for 3 months after discharge. The patient reported no discomfort, such as headache or epilepsy, during the follow-up time.

## Discussion

Meningioma has an incidence rate of 16%–23%, accounting for 30% of intracranial tumors, making it the most common type of intracranial tumor after glioma. The incidence increases with age and is often seen in people aged 60–70 years. The female-to-male ratio is 2:1, which may be associated with progesterone and PR-positive meningioma. The absence of NF2 is a risk factor, and ionizing radiation (IR) exposure also plays a role as well (11). It has been reported that only 1.1% of patients with meningioma have a coexisting aneurysm (8).

The initial diagnosis of an intracranial occupancy or aneurysm is not difficult to make nowadays with supplement examination methods. Modern imaging techniques such as MRI, CT, and DSA can easily detect occupancies such as glioma and meningioma and ventricular abnormalities like aneurysms. Meningioma presents isointensity on T1-weighted sequences and hyperintensity on T2-weighted and fluid-attenuated inversion recovery sequences. In addition, the “dural-tail sign” can also suggest the presence of a meningioma, which highly matches with the imaging of our patient (12). MRA has high sensitivity for intracranial aneurysms, but it is still difficult to investigate the presence of small or occultly located aneurysms. In our case, the A2 aneurysm can only be found when the DSA is performed.

In our case, all the aneurysms of the patient were located in the edema area originating from the meningioma in the frontal lobe, which creates the possibility to perform resection and clipping in one surgery. In 1994, Stevenson et al. presented a case report of a patient with multiple aneurysms, multiple meningiomas, and multiple subcutaneous angiolipomas. They performed two surgeries on this patient due to the anatomical position of the meningiomas and aneurysms. The gap between surgeries was 5 months. The similarity is that we both resected the meningioma in the frontal lobe and clipped the aneurysm at the end of the therapy. The difference between these two cases is that in preoperative time, MRI was performed on the patient. According to the imaging result, the location and the border of the tumor were clearer than those in the CT scan. What needs to be mentioned here is that multiple surgical targets may make intraoperative neuronavigation inaccurate; real-time navigation like intraoperative ultrasound will be reliable for multiple existing brain tumors or the brain shift (13). We did not perform the surgery on the occupancy placed in the right temple bone; instead, we chose to observe and follow up. In their case, during craniotomy, an en plaque meningioma was also found and they resected it (5).

We retained the abnormal occupancy under the temple bone because it is smaller than 1.5 cm, so we chose to follow up, considering both the size of the occupancy and the patient’s family income. In the 3-month follow-up, the occupancy under the temple bone seems asymptomatic, which makes our plan reasonable. In their research, no symptom has been reported during the 5 months of follow-up. Still, the second stage of surgery was performed on the patient.

The course of the seizure after the surgery was unknown. According to the situation of the patient’s MRI scan, the range of edema around the meningioma was large, and 3 days after the resection, the CT scan still showed a low-density area around the original position of the occupancy. These together could lead to the disturbance of neuroelectrophysiology, which could explain the single seizure after the surgery (14). The tumor volume seems to have no significant effect on causing the postoperative seizure (15). After giving sodium valproate regularly, the seizure never happened before the discharge and in the 3-month follow-up.

No research has proved the mechanistic correlation between a meningioma and an aneurysm. In previous research, patients with meningiomas have a higher chance of suffering from intracranial vascular abnormalities (including intracranial aneurysm) due to hypertension and tumor volume (2). According to Fischer et al. in their research, the incidence of meningiomas and aneurysms occurring together was 1.1%, which is lower than the average incidence of meningiomas and aneurysms alone. In their study, they hypothesized that higher-grade meningiomas alter the hemodynamics of the blood-supplying arteries and, therefore, lead to aneurysm formation; there is no relationship between a meningioma and a cerebral aneurysm, and the coexistence of brain tumor and vascular malformation is just an accident (1).

Further investigation is needed to determine the relationship between tumor volume and aneurysm formation. In our case, we can assume that the increased blood supply in the tumor changes the hemodynamics of the anterior circulation, thus causing aberrant flow, leading to mechanical overload and a shift in tensile forces and forming an aneurysm (4, 16). We can also assume that edema caused by the obstruction of venous reflux due to the skull base meningioma has no role in the formation of aneurysms, but it surely leads to a seizure postoperation. It has been reported that the appearance of a single meningioma and a single aneurysm seems to be on the same side or nearby, making this hypothesis more convincing (17, 18). In one case reported by Tanaka et al., after the resection of the frontal lobe meningioma, the aneurysm in the internal carotid artery (ICA) immediately ruptured (19).

However, this explanation cannot cover all cases, as there are still meningiomas located in the area where aneurysms are located on the other side (6). In our case, the distant occupancy in the right temple bone appears asymptomatic in a 3-month follow-up. So, further investigation and follow-up are needed.

There is still room for improvement in terms of diagnosis and treatment. According to the 2021 European Association of Neuro-Oncology (EANO) guidance on the diagnosis and management of meningioma, although positron emission tomography (PET) is not included in the standard treatment procedure, it could be an option to distinguish the tumor from healthy tissue (12). Considering the patient’s income, we finally decided not to perform PET on the patient. Instead, MR sequences and CT images are sufficient to show the basic imaging features of the tumor and to decide on the initial plan of surgery. MRA has its advantage in showing aneurysms, but in our case, the A3 segment aneurysm is only reported in the DSA scan, which informs that DSA still places itself as the “gold standard” for the intracranial aneurysm. From another perspective, DSA seems not to be necessary for this patient; the size of the aneurysm placed in the A2 segment is limited. Considering that the patient does not have hypertension or other risk factor, it will be just fine not to check it.

Stereotactic radiosurgery (SRS) like Gamma knife is also a possible way to treat meningiomas under the right temple bone and the possible residual recurrence of the falx and skull base meningiomas. First, for those meningiomas that have infiltrated the vessels, the skull base, or the cranial nerves, SRS reduces the residual (20, 21). Second, after the surgery, the patient did not complain of the headache. SRS is also effective for nonsymptomatic meningiomas (21). The same field of vision of excision makes the clipping of the three aneurysms seem like an “accident”, but this can lead to the treatment presented here—if the aneurysm is located in close proximity to the optic nerve after the removal of the skull base meningioma, then clipping is reasonable, even if the aneurysm is in the range of the edema area caused by the tumor (6). For the meningioma, it is also good to observe whether the tumor size is smaller than 10 mm or it does not disturb the neurofunction (11).

## Conclusion

It is rare for multiple meningiomas and multiple aneurysms to be present in a single patient. Considering the possible association between tumor and ventricular abnormality, this case could provide a good indication and reference for subsequent clinical and scientific researchers in terms of histology, diagnosis, treatment, and prognosis. Further studies need to focus on the mechanism of the association between intracranial meningiomas and aneurysms. We hope our research can play the role of a supplement to guide the treatment of coexisting meningiomas and aneurysms.

## Data Availability

The original contributions presented in the study are included in the article/Supplementary Material, further inquiries can be directed to the corresponding author.
